# The occurrence of depressive symptoms in obese subjects starting treatment and not seeking treatment for obesity

**DOI:** 10.1007/s40519-018-0578-y

**Published:** 2018-09-27

**Authors:** Wojciech Gruszka, Katarzyna Wyskida, Aleksander J. Owczarek, Ewa Jędrusik, Nasser Alraquayee, Mateusz Glinianowicz, Monika Bąk-Sosnowska, Jerzy Chudek, Magdalena Olszanecka-Glinianowicz

**Affiliations:** 1 Health Promotion and Obesity Management Unit, Department of Pathophysiology, Medical Faculty in Katowice, Medical University of Silesia, Katowice, Poland; 2 Pathophysiology Unit, Department of Pathophysiology, Medical Faculty in Katowice, Medical University of Silesia, Medyków Street 18 20, 40-752 Katowice, Poland; 3 Department of Statistics, School of Pharmacy with the Division of Laboratory Medicine in Sosnowiec, Medical University of Silesia, Katowice, Poland; 4 Department of Psychology, Chair of Social Sciences and Humanities, School of Health Sciences in Katowice, Medical University of Silesia in Katowice, Katowice, Poland; 5 Department of Internal Medicine and Oncological Chemotherapy, Medical Faculty in Katowice, Medical University of Silesia, Katowice, Poland

**Keywords:** Obesity, Depressive symptoms, Treatment

## Abstract

**Introduction:**

The psychological profile of obese people deciding to start obesity treatment may be different from those not choosing to seek the therapy. Previous studies have shown a higher incidence of depression in obese than in normal weight people. However, data are lacking concerning the occurrence of depressive symptoms and their severity in obese subjects who do or do not decide to start treatment for obesity. Therefore, the aim of this study was to evaluate the prevalence and severity of depressive symptoms among obese people starting treatment and not seeking treatment for obesity.

**Materials and methods:**

Enrolled subjects were 331 adults (241 women, 90 men), including 193 obese subjects starting treatment for obesity (46.8 ± 13.2 years, BMI 37.6 ± 5.5 kg/m^2^) and 138 obese volunteers never seeking treatment for obesity (44.3 ± 12.5 years, BMI 34.7 ± 4.3 kg/m^2^). Depression levels were determined using the Beck Depression Inventory (BDI).

**Results:**

The level of depression was significantly higher among those starting treatment for obesity than those never seeking treatment for obesity (13.2 ± 9.2 vs. 9.5 ± 7.9 points; *p* < 0.001). This difference was statistically significant in women (14.4 ± 9.2 and 11.0 ± 8.2 points, respectively; *p* < 0.01), but not in men (7.2 ± 6.4 and 7.3 ± 7.1 points, respectively; *p* = 0.95). There were more women with moderate/severe depressive symptoms in the group starting treatment than in the group not seeking treatment for obesity (44.7 and 24.4%, respectively). No such difference was observed in men.

**Conclusions:**

Obese subjects, especially women, with depressive symptoms are more likely to start treatment for obesity.

**Level of evidence:**

Level III, case-control analytic study.

## Introduction

Obesity and its complications remain a significant public health concern. In 2007 it was estimated that the prevalence of obesity has tripled in developing countries in the past 20 years mainly due to unhealthy eating habits and sedentary lifestyle [1]. In addition, more recent studies indicate obesity as one of the leading causes of disability and death, affecting not only adults, but also children and adolescents [2]. The contribution of psychological factors to obesity development remains unclear.

An increased incidence of depression predisposing to obesity development has been reported by several previous studies, including our own [3–5]. Previously we have observed mild and moderate/severe depressive symptoms in 38% and 49% subjects starting participation in obesity treatment program, respectively. In addition, we showed that the prevalence of depressive symptoms was higher among women and was most severe among the morbidly obese [5]. Moreover, data from several studies suggest that mental health conditions, especially depression and binge eating disorder, are common in patients treated with bariatric surgery [6]. Currently there are no guidelines for assessment of depression and its symptoms in patients with obesity. However, mental health evaluation, especially before bariatric surgery procedures is recommended [7].

Depression seems to be one of the predisposing factors for obesity development, and vice versa [4]. Previously published studies suggest that depressive symptoms are linked with higher consumption of sweet foods [8, 9] but lower consumption of fruits and vegetables [9]. The occurrence of depressive symptoms is also associated with lower physical activity levels [10, 11]. Depression might attribute to obesity via sleep disturbances [12] and gut microbiota pathology [13]. As mentioned above, obesity may also lead to the development of depression [14, 15]. Discrimination and social stigmatization by other people [16], lower socioeconomic status [17, 18], and body image dissatisfaction [19] are considered as the main causative factors for depression in obese individuals.

With concern to depressive symptoms most of the studies up to now considered obese people as homogenous group. However, it has been suggested by Fitzgibbon et al. [20] that psychological profiles among obese people, especially between those opting for treatment for obesity and those who are not seeking treatment, are heterogeneous. Among obese seeking treatment for obesity more frequently psychopathology and binge eating disorder were observed than in both obese group not seeking treatment and normal weight. However, in both obese groups more frequently were observed symptoms of distress, negative emotional eating, overeating, difficulty in resisting temptation than in normal weight group [20]. Furthermore, Strømmen et al. [21] showed clear difference between obese individuals selecting particular type of obesity intervention, in their study, surgery or lifestyle interventions. It was also indicated that obese individuals seeking lifestyle interventions were more severe obese and had more severe depressive symptoms [22].

It can be assumed that most or even all obese or overweight subjects in the years-lasting course of their condition try to lose weight. These attempts can be based on knowledge acquired in many ways. However, not all are looking for obesity treatment supervised by medical doctors or other professionals. Those who are seeking obesity treatment can represent some particular features different than subjects not-seeking for treatment. What is more, depressive symptoms in obesity could be a reason in itself to seek treatment for obesity in the hope of feeling better after losing weight. It cannot be excluded that in this case the obese expect treatment for both obesity and depression. On the other hand, depressive symptoms can be also a factor that may lead to delay in seeking treatment for obesity.

Factors motivating to seek treatment for obesity described before included family support [23], quality of life [24] and willingness to improve appearance [25]. However, taking into account increasing prevalence of overweight and obesity [1, 2], finding characteristics for people not prone to take steps towards weight reduction should be of high priority in public health. The improvement in help-seeking rate for obese person should have potential benefits in the struggle with the obesity epidemic.

Therefore, the aim of the present study was to assess depression levels in obese subjects starting treatment for obesity and never seeking treatment for obesity.

## Materials and methods

Enrolled subjects were 331 adults [241 (72.8%) women], including 193 (53.8%) obese subjects starting treatment for obesity (46.8 ± 13.2 years, BMI 37.6 ± 5.5 kg/m^2^) and 138 (46.2%) obese volunteers never seeking treatment for obesity and showing no interest in such therapy (44.3 ± 12.5 years, BMI 34.7 ± 4.3 kg/m^2^). The 6-month group treatment consisted of regular meeting with the physician, dietitian, psychologist and physiotherapist (every 2 weeks). No pharmacological interventions were applied. This treatment was aimed to obtain 5–15% body mass reduction in this period, as recommended [26]. The patients did not have to pay for it. The obese volunteers were recruited by co-authors which are physicians in their out-patient’s department (the following two questions were posed: Have you ever tried professional treatment against obesity? Are you interested in this kind of treatment?). The recruitment rate was 82%. The reasons of visits were various, excluding weight problems. Basic characteristics (age, body mass, BMI and the Beck Depression Inventory—BDI scores) of participants with the division into the gender are presented in Table 1.

**Table 1:** Study groups characteristics

	Group starting treatment for obesity			Group not seeking treatment for obesity		
Group:	All *N* = 193 (58.3%)	Men *N* = 34 (17.6%)	Women *N* = 159 (82.4%)	All *N* = 138 (41.7%)	Men *N* = 56 (40.6%)	Women *N* = 82 (59.4%)
Age (years)	46.8 ± 13.2 (18–73)	49.5 ± 13.0 (18–68)	46.2 ± 13.2 (18–73)	44.3 ± 12.5 (20–76)	40.6 ± 14.4 (20–76)	46.8 ± 10.5 (23–69)
Body mass (kg)	101.2 ± 17.8 (66–159)	115.6 ± 17.5 (83–146)	98.1 ± 16.3 (66–159)	98.1 ± 15.6 (67–139)	105.7 ± 14.6 (79–139)	92.9 ± 14.1 (67–139)
BMI (kg/m^2^)	37.6 ± 5.5 (30.0–66.2)	37.8 ± 4.3 (31.3–48.5)	37.6 ± 5.7 (30.0–66.2)	34.7 ± 4.3 (30.0–50.6)	34.2 ± 3.9 (30.0–46.5)	35.1 ± 4.5 (30.0–50.6)
BDI total (pts)	13.1 ± 9.2 (0–48)	7.2 ± 6.4 (0–29)	14.4 ± 9.2 (1–48)	9.5 ± 7.9 (0–37)	7.3 ± 7.1 (0–36)	11.0 ± 8.2 (0–37)
BDI corr. (pts)	9.3 ± 7.3 (0–36)	4.8 ± 5.2 (0–20)	10.3 ± 7.4 (0–36)	6.7 ± 6.0 (0–27)	5.3 ± 5.5 (0–25)	7.7 ± 6.2 (0–27)

Mean ± standard deviation and range

The inclusion criteria were age over 18 years, obesity (BMI ≥ 30 kg/m^2^), stable body mass at least 3 months before the enrollment, and a history of obesity lasting for at least some years. The exclusion criteria were secondary obesity (endocrine disorders like Cushing’s syndrome and genetic disorders like Turner or Prader–Willi syndromes) and history of mental illness (lifetime bipolar disorders, schizophrenia and current substance dependence) as well as anxiety and eating disorders and use of antidepressant medication. Above data falling partially outside the standard medical interview was obtained on the basis of own questionnaire.

Due to low BMI, mental illness (recorded in patient’s history) or incorrect filling or a substantial amount of missing data in BDI questionnaire 9.7% of initial number of subjects were excluded. Finally, as mentioned above a study group consists of 331 people, including 241 women (72.8%) and 90 men (27.2%).

The study was approved by the Bioethical Committee Medical University of Silesia, and all subjects gave informed consent for participation in the study.

Body mass (without shoes, in light clothing, using the certified electronic RADWAG scales, with an accuracy of 0.1 kg) and height (in an upright standing position, without shoes, with an accuracy of 0.5 cm, using an integral part of the RADWAG scales) were measured. BMI was calculated using the standard formula. Assessment of nutritional status was based on BMI according to WHO criteria: underweight < 18.5 kg/m^2^, normal weight 18.5–24.9 kg/m^2^, overweight 25–29.9 kg/m^2^, obesity ≥ 30 kg/m^2^ [27].

The depression level was determined using the self-reported Polish adaptation of the full 21-item version of Beck Depression Inventory (BDI) [28]. Translation and validation of this questionnaire were done by Parnowski and Jernajczyk [29]. Both English and Polish versions perform well psychometrically [28, 29]. BDI was administered as hard copy. Items consist of 4 statements scored 0–3, with higher scores indicating increasing symptom severity. Respondents were instructed to describe the way they have been feeling during the past 2 weeks. Total BDI scores were calculated. On that basis, study subjects were allocated to subgroups: without symptoms of depression (0–9 pts.), with mild symptoms of depression (10–15 pts.), or moderate/severe symptoms of depression (16 pts. or more). The cutoff score of ≥ 16 pts. was set following Udo et al. [30], as this value demonstrated moderate discriminating accuracy.

As the next step, somatic / physical items from BDI were excluded (loss of energy, sleep problems, irritability, appetite problems, concentration, fatigue, loss of interest in sex), following the previous authors [31], as these can overlap with the symptoms of obesity rather than being due to depression [31, 32]. The scores defined as corrected BDI scores were calculated.

### Statistical analysis

Statistical analysis was performed using the STATISTICA 10.0 PL software (Cracow, Poland). The results were presented as mean ± standard deviation for data in interval scale and as percentages for data in nominal and ordinal scales. The assessment of normality was based on the Shapiro–Wilk test and quantile–quantile plot. To compare groups of subjects the *χ*^2^ tests and analysis of covariances ANCOVA (with age and BMI as independent interval variables, treatment and gender as independent nominal variables) with contrast analysis were used. Contrast comparison enclosed: (1) comparison between men and women starting treatment and men and women not seeking treatment and (2) comparison between group starting treatment and not seeking treatment in men and women separately. Results were considered as statistically significant with a *p* value less than 0.05.

## Results

**Fig. 1:**
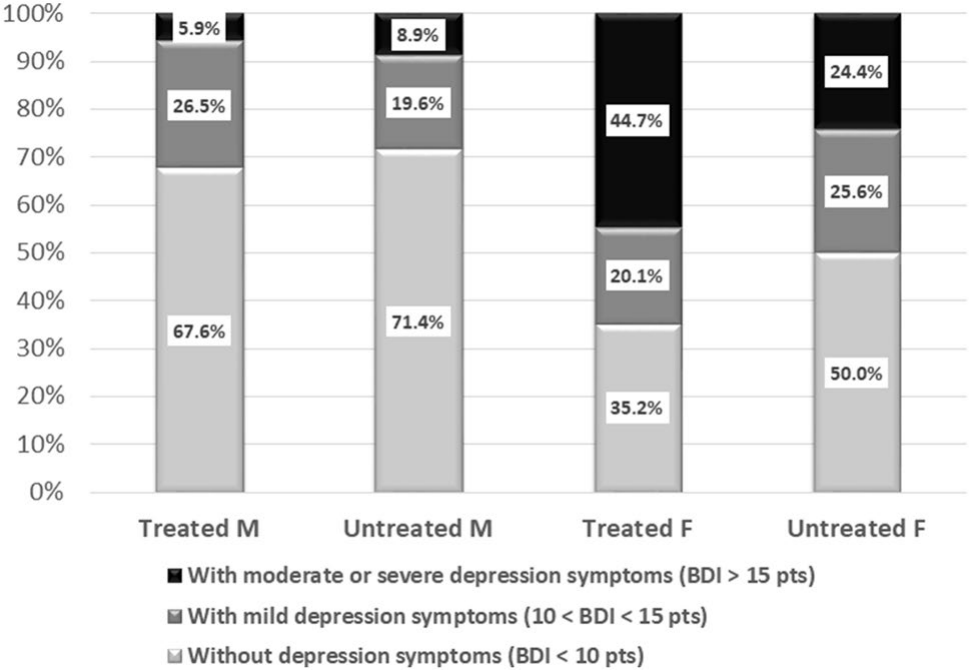
Distribution of depressive symptoms severity for men and women in group starting treatment and group not seeking treatment for obesity (*M* male, *F* female)

In the group of obese subjects starting therapy for obesity, there were 73 subjects (37.8%) with moderate/severe depressive symptoms, 41 subjects (21.2%) with mild symptoms, and 79 subjects (41.0%) without symptoms. In the group of obese volunteers not seeking treatment for obesity and showing no interest in such therapy, there were 25 subjects (18.1%) with moderate/severe depressive symptoms, 32 subjects (23.2%) with mild symptoms, and 81 subjects (58.7%) without symptoms. There was statistically significant difference between those two groups in depressive symptoms distribution (*χ*^2^ = 15.95, *p* < 0.001). In the group of obese subjects starting a treatment for obesity, significantly more subjects had moderate/severe depressive symptoms than in the group not seeking treatment for obesity. Figure 1 shows the distribution of moderate and severe depressive symptoms for men and women in the group starting treatment and the group not seeking treatment for obesity. There were significant differences in the distribution of mild and moderate/severe depressive symptoms in women (*χ*^2^ = 9.55; *p* < 0.001), but not in men (*χ*^2^ = 0.74; *p* = 0.691).

Obese subjects starting therapy tended to be older (*p* = 0.08) and had greater BMI by 2.9 km/m^2^ (*p* < 0.01).

### Total and corrected BDI scores

**Fig. 2:**
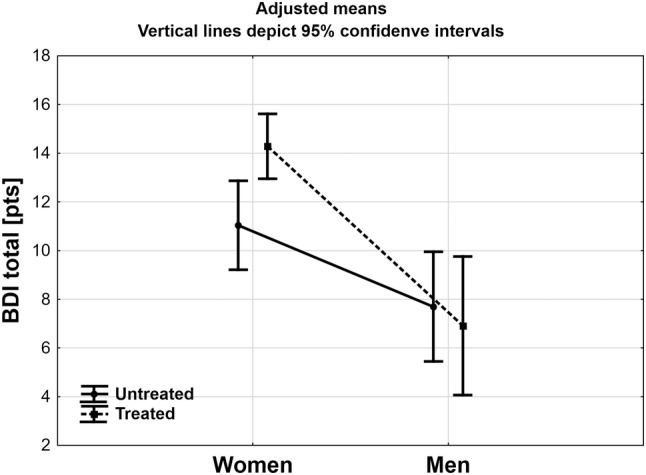
Mean total score of Beck Depression Inventory in studied groups

Results of ANCOVA analysis are presented in Table 2. Adjusted R for the model was 0.329 with *F* = 8.997 (*p* < 0.001). Female gender proved to be the only significant factor increasing the BDI values. There was also a tendency to statistical significance in interaction between gender and treatment. Results of adjusted means (for mean age: 45.8 years and mean BMI: 36.2 kg/m^2^) are presented in Fig. 2. Women starting treatment for obesity had significantly higher values of BDI then those not seeking treatment for obesity (*p* < 0.01), while there was no such difference in men (*p* = 0.68). In both starting treatment and not seeking treatment group women had significantly higher values of BDI then men (*p* < 0.001 and *p* < 0.05 respectively).

**Table 2:** Results of ANCOVA analysis

	BDI total (pts)						BDI corrected (pts)					
	$\beta$	SE (β)	− 95% β	+ 95% β	$p$	${\eta ^2}$	$\beta$	SE(β)	− 95% β	+ 95% β	$p$	${\eta ^2}$
Age (years)	0.040	0.036	− 0.031	0.112	0.27	0.196	0.004	0.029	− 0.052	0.061	0.88	0.052
Treatment	− 0.616	0.558	− 1.713	0.481	0.27	0.196	− 0.445	0.442	− 1.314	0.425	0.32	0.171
BMI (kg/m^2^)	0.085	0.092	− 0.096	0.267	0.36	0.152	0.051	0.073	− 0.093	0.195	0.48	0.107
Female gender	2.679	0.537	1.624	3.735	< 0.001	0.999	1.992	0.425	1.155	2.829	< 0.001	0.997
Female gender × Treatment	− 1.006	0.543	− 2.074	0.062	0.06	0.455	− 0.807	0.430	− 1.654	0.040	0.06	0.107
Const	5.065	3.613	− 2.042	12.172	0.16	0.287	4.980	2.864	− 0.654	10.614	0.08	0.411

**Fig. 3:**
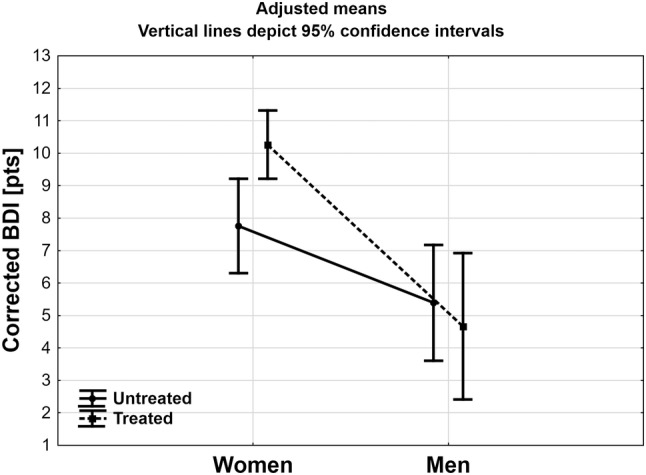
Mean score of Beck Depression Inventory corrected in studied groups

The results for corrected BDI scores ANCOVA analysis are presented in Table 2. Adjusted R for the model was 0.300 with *F* = 7.527 (*p* < 0.001). Additionally, in this case the female gender proved to be the only significant factor increasing the corrected BDI values. Moreover, there was a tendency to statistical significance in interaction between gender and treatment. Results of adjusted means are presented in Fig. 3. Women starting treatment for obesity had significantly higher values of corrected BDI then those not seeking treatment (*p* < 0.01), while there was no such difference in men (*p* = 0.62). In both starting treatment and not seeking treatment for obesity group women had significantly higher values of BDI then men (*p* < 0.001 and *p* < 0.05 respectively).

## Discussion

The present study seems to be one of the first that assessed the differences between obese subjects starting treatment and not seeking treatment for obesity. Our results partially confirm the hypothesis that the depressive disorders of obese subjects are heterogeneous. Obese subjects with depressive symptoms were more likely to start treatment for obesity. This relation was also observed after excluding from total BDI scores points representing somatic symptoms. However, higher depression levels were found in obese women, only. Therefore, it seems that some women may seek out treatment for their psychological distress rather than the obesity, or their depressive symptoms may motivate them to seek treatment for obesity.

It should be noted that BMI did not influence depressive symptoms levels. This is partially contrary to our previously published observation that prevalence and severity of depressive symptoms increase with degree of obesity [4]. Additionally, in accordance with other studies, age was a factor associated with greater prevalence of depressive symptoms, although its impact was relatively small [33].

The nature of the causal relationship between obesity and depression remains unclear. It seems that a bidirectional relationship is possible. The last meta-analysis carried out by Faith et al. [4] indicated that the path of “obesity leading to depression” seems to be more common in the general population. In addition, considering our results, a further pathway in women—“depression leads to seeking obesity treatment”—is likely.

This hypothesis is contradictory to our previously published results, showing that depressive symptoms levels did not influence the effectiveness or the duration of continuation of a obesity treatment program [34–36]. The factors responsible for the above phenomena are probably more complex, and not all predictors have been identified yet. It should be emphasized that, to our knowledge, this area of behavior of obese people has been poorly investigated. It cannot be excluded that apart from symptoms of depression, many other personality features and emotional disturbances influence this decision. Moreover, numerous external factors, such as pressure from family or friends may also play an important role.

There is a great need for a more detailed assessment of the features of obese people who are starting treatment and not seeking treatment for obesity. Easy to use screening assessment tools should be found to select obese individuals neither seeking obesity treatment, nor perceiving their obesity per se as a health problem. These personality features and emotional disturbances need to be modified, which may encourage obese people to attempt weight reduction. On the other hand, presented results can also imply that clinicians should screen obese female patients (who seek therapy for their obesity) for depression. In case of positive screening result, refer these patients to the psychiatrist.

The main limitation of the presented study is the relatively small number of male subjects. It should be emphasized that in our study only the Beck Depression Inventory was used to assess depressive symptoms. The BDI determines only the level of symptoms of depression, it is not a tool to diagnose depression according to the nine criteria from the Diagnostic and Statistical Manual of Mental Disorders, Fifth Edition [37]. To exclude somatic/physical symptoms we used BDI factor structure proposed by Thombs et al. [31], however, different factors solutions are reported in the literature [30, 38, 39]. Some previous studies used different cutoff scores for BDI [39, 40], what makes results difficult to compare. As suggested before the optimal cutoff for any screening test seems to depend on context that it will be used in [30, 41]. That’s why we exclude a group with “moderate” depressive symptoms believing that the risk of depression development in this group is elevated and this subjects need at least check-ups. However, the position of cutoff value seems not to influence observed phenomenon, what was proved by analysis of mean values of BDI scores. What is more, there is lack of cutoff values for BDI excluding somatic items, so that we decided to present this data only using mean values. Therefore, further studies should focus on male obese subjects and other socio-demographic and psychological factors that predispose them to undertake treatment for obesity. In addition, the limitation of this study are also the lack of assessment of personality features and external factors such as pressure from family that may influence the decision of seeking obesity treatment. Another limitation is cross-sectional design. However, our previously published results shown that the depression level at baseline did not influence the effectiveness of the group weight reduction program [35].

## Conclusion

Obese subjects, especially women, with depressive symptoms are more likely to start treatment for obesity.
